# A systematic review of the cost-effectiveness of renal replacement therapies, and consequences for decision-making in the end-stage renal disease treatment pathway

**DOI:** 10.1007/s10198-022-01478-2

**Published:** 2022-06-18

**Authors:** Ellen Busink, Dana Kendzia, Fatih Kircelli, Sophie Boeger, Jovana Petrovic, Helen Smethurst, Stephen Mitchell, Christian Apel

**Affiliations:** 1 Health Economics, Market Access & Political Affairs, Fresenius Medical Care, Else-Kröner-Straße 3, 61352 Bad Homburg, Germany; 2 Global Medical Information & Education, Fresenius Medical Care, Bad Homburg, Germany; 3 Mtech Access Ltd, Bicester, UK

**Keywords:** Systematic review, Renal replacement therapy, Patient pathway management, Patient choice, Economic evaluation, Healthcare policy, I110, I120

## Abstract

**Objectives:**

Comparative economic assessments of renal replacement therapies (RRT) are common and often used to inform national policy in the management of end-stage renal disease (ESRD). This study aimed to assess existing cost-effectiveness analyses of dialysis modalities and consider whether the methods applied and results obtained reflect the complexities of the real-world treatment pathway experienced by ESRD patients.

**Methods:**

A systematic literature review (SLR) was conducted to identify cost-effectiveness studies of dialysis modalities from 2005 onward by searching Embase, MEDLINE, EBM reviews, and EconLit. Economic evaluations were included if they compared distinct dialysis modalities (e.g. in-centre haemodialysis [ICHD], home haemodialysis [HHD] and peritoneal dialysis [PD]).

**Results:**

In total, 19 cost-effectiveness studies were identified. There was considerable heterogeneity in perspectives, time horizon, discounting, utility values, sources of clinical and economic data, and extent of clinical and economic elements included. The vast majority of studies included an incident dialysis patient population. All studies concluded that home dialysis treatment options were cost-effective interventions.

**Conclusions:**

Despite similar findings across studies, there are a number of uncertainties about which dialysis modalities represent the most cost-effective options for patients at different points in the care pathway. Most studies included an incident patient cohort; however, in clinical practice, patients may switch between different treatment modalities over time according to their clinical need and personal circumstances.

Promoting health policies through financial incentives in renal care should reflect the cost-effectiveness of a comprehensive approach that considers different RRTs along the patient pathway; however, no such evidence is currently available.

**Supplementary Information:**

The online version contains supplementary material available at 10.1007/s10198-022-01478-2.

## Introduction

**Fig. 1:**
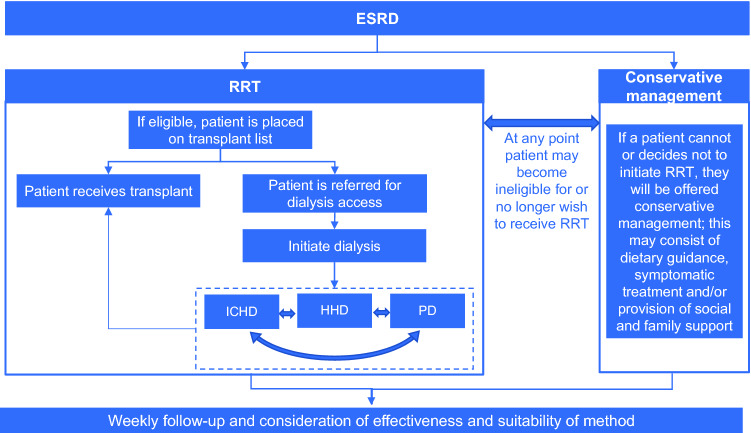
Treatment algorithm for ESRD management. *ESRD* end-stage renal disease, *HHD* home haemodialysis, *ICHD* in-centre haemodialysis, *PD* peritoneal dialysis, *RRT* renal replacement therapy

Chronic kidney disease affects up to 16% of the adult population globally, and is associated with poor outcomes upon progression to end-stage renal disease (ESRD) [1]. Typical ESRD patients commencing renal replacement therapy (RRT) are inherently in rapidly declining health, are relatively old (median age of 64.0 years in the UK in 2018 [2]), have several common comorbid conditions, and span socioeconomic classes [3, 4]. While kidney transplant is regarded as the optimal RRT [5], donor availability and patient eligibility are limiting factors in practice [6–8]. For this reason, the majority of ESRD patients undergo dialysis. Figure 1 provides an overview of the current pathway for management of ESRD. The predominant dialysis modalities are haemodialysis (HD; which can be undertaken in-centre [ICHD] or at home [HHD]), and peritoneal dialysis (PD), which is generally undertaken at home [9–11]. These techniques, though all considered efficacious, are distinct in terms of their practical application. Eligibility for HHD and PD is dependent on the willingness and ability of patients to manage their own care, a suitable home environment in which to perform these procedures, and is subject to certain medical requirements [12, 13]. Patients may require a number of modality changes over their lifetime on RRT [14–16]. For example, many patients who receive PD may require a switch to a HD treatment modality after 2–3 years. Residual renal function is extremely important in PD-treated patients and needs to be monitored closely [17]. In addition, the peritoneal membrane is a living structure that is prone to alteration over time, for example due to dialysis fluid characteristics (e.g. pH, osmolarity, glucose degradation products, and bioincompatibility) and peritonitis, which lead to loss of the membrane properties that enable efficient treatment [18]. In clinical practice, that means that peritoneal membrane performances (water and solute mass permeability) should be monitored periodically to detect early deterioration [19]. In the case of dialysis inadequacy, a switch to another form of RRT should be considered [20]. Therefore, offering the best mode of dialysis at the right time for each patient is crucial [15, 16].

Appropriate decision-making is fundamental in renal care, for which effective implementation and continuation of care is crucial to prolonging survival and improving a patient’s quality of life. Furthermore, RRT is a significant burden on finite healthcare resources; across Europe RRT accounts for 2% of healthcare expenditure for 0.1% of the population, with an estimated total cost of €15 billion per year [21]. In general, economic evaluations are used to provide high-level predictions of whether an intervention may be of value to a system relative to established practice. Such analyses are often used to support decision-making at a national level, which subsequently influences local policies and individual decisions. However, the cost-effectiveness framework generally represents the use of an intervention at a particular point in care for the ‘average’ patient. As such, traditional methods are restrictive in accommodating the complexities of the actual life of a patient with a chronic disease such as ESRD.

National guidance and public health policies have emerged that recommend or incentivise specific dialysis modalities based upon conclusions of cost-effectiveness [22–25]. The aim of this analysis was to systematically identify and assess existing cost-effectiveness analyses of dialysis modalities and to understand whether the current published data reflect the complexities of the ESRD patient pathway.

## Methods

A systematic literature review (SLR) was conducted to identify published cost-effectiveness studies comparing two or more distinct dialysis techniques in the management of ESRD. Since renal transplant is accepted to be the superior mode of RRT for clinically suitable patients [26], studies for which the objective was to assess the cost-effectiveness of transplant were excluded from this research; however, studies that considered transplant as one of several treatment options have been included (with results relevant to transplant not reported). The following databases were searched: Embase, MEDLINE, EBM Reviews and EconLit. The review was initially conducted between 2005 and March 2020 and was subsequently updated to capture the most recently published studies up to July 2021. The review was conducted according to the guidelines of the Preferred Reporting Items for Systematic Reviews and Meta-Analyses (PRISMA) [27]. The full search strategy is presented in Supplementary Information section S1 and S2.

Abstracts and full texts were screened by two reviewers based on the criteria described in Table 1. Studies that were not in English or for which no full text was available were excluded. Only studies published since and using direct costs reflective of 2005 onwards were considered, to ensure the relevancy of discussions to current practice. Studies that did not comprise a comparison of distinct dialysis methods (e.g. HD vs haemodiafiltration; continuous ambulatory peritoneal dialysis [CAPD] vs automated peritoneal dialysis [APD]) were also excluded. Therefore, the only comparisons considered in this analysis were: ICHD vs PD and/or HHD. Extraction of relevant variables and assessment of bias [28] for all included studies were performed by two reviewers, with any disagreements resolved by discussion and/or additional referees.

**Table 1:** Population, intervention, comparison, outcomes and study design for systematic review of published cost-effectiveness analyses of dialysis for ESRD

	Inclusion criteria
Population	Patients with ESRD
Intervention	Any form of dialysis
Comparators	
Outcomes	Model design, costs, health outcomes (e.g. QALYs, LYG), cost-effectiveness estimates (e.g. ICER)
Study design	Cost-effectiveness analysis, cost–utility analysis

*ESRD* end-stage renal disease, *ICER* incremental cost-effectiveness ratio, *LYG* life years gained, *QALY* quality-adjusted life-year

## Results

### Characteristics of included studies

**Fig. 2:**
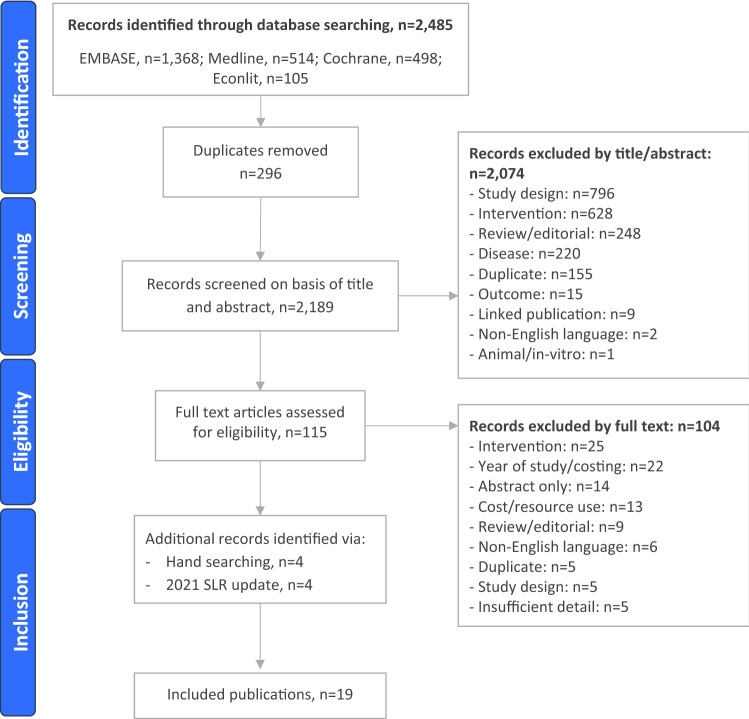
PRISMA flow diagram

The initial search identified 2189 records and the update identified 189 records. In total, 19 publications comparing distinct modalities were eligible for inclusion (Fig. 2). The completed quality assessment of studies can be found in Supplementary Table 1.

### Populations and interventions considered

A summary of the methodology of the included studies is presented in Table 2. All included studies considered adult patients undergoing dialysis for ESRD, while two studies restricted their patient populations to those > 60 years of age [29, 30]. The vast majority of studies (13/19) considered an incident population (i.e. patients who were starting dialysis for the first time) [29–41], while 6/19 studies did not specify, or considered a prevalent or mixed incident/prevalent population [42–47]. One study presented outcomes for subgroups of patients with or without diabetes [30]. In total, ten studies compared the cost-effectiveness of ICHD with PD only (or also with transplant, which is not a treatment option of consideration for this analysis) [30, 31, 33, 34, 37, 39, 40, 42, 45, 47], while a further three studies compared HD with PD only, but the location of HD (in centre or at home) was not reported [38, 41, 44]. Three studies compared ICHD with HHD only [32, 36, 43], two studies compared ICHD with HHD and PD [29, 35], while one study considered HD administered in-centre, at a satellite centre, as self-care or at home compared with PD [46]. Across the studies that considered HHD, the duration and frequency of administration varied from conventional (3 sessions × 4 h per week) to intensive home haemodialysis (iHHD; every other day, 5 times per week or nocturnal HD) [29, 32, 35, 36, 43, 46]. Eight studies looked at the potential impact of varying PD uptake rates from existing local levels [30, 34, 35, 37–39, 45]; one of these studies also looked at the impact of increasing HHD uptake from the existing treatment mix [35].

**Table 2:** Summary of methods of published dialysis cost-effectiveness studies

Study^a^, country	Population (mean patient age)	Interventions	Model type, perspective (reference year)	Time horizon, discounting	Clinical data (source)	Utility elicitation method	Utilities	Cost data (source)	Direct non-medical or indirect costs incorporated?
Afiatin, 2017 [30], Indonesia	Incident patients > 18 years with ESRD undergoing RRT (NR)	ICHD-first policy PD-first policy SoC	Markov, payer and societal (2015)	Lifetime, 3% for costs and outcomes	Survival (national registry), probability of peritonitis or vascular access complication (published sources), probability of switching from HD to PD or from PD to ICHD (published source)	EQ-5D-3L using the Thai value set	PD without/with complication: 0.82/0.31 HD without/with complication: 0.70/0.37	SoC, initial and maintenance ICHD and PD costs, annual ICHD and PD complication costs (billing costs)	Direct non-medical costs (including travel, food, accommodation), indirect non-medical costs including income loss and utility costs (questionnaire)
Bayani, 2020 [38], Philippines	Incident adult patients with chronic ESRD requiring RRT (52 years)	ICHD PD Transplant	Markov, provider and societal (2019)	Lifetime horizon. 3% for costs and outcomes	Survival (national registry and database)	EQ-5D using the Philippines value set	Twice weekly HD: 0.667 Thrice weekly HD: 0.697 PD: 0.779 Post-kidney transplant: 0.911	Direct costs associated with all treatment options, societal costs of PD/HD, annual post-transplant maintenance, reimbursement (published case rates, microcosting analysis, questionnaire survey)	Annual societal cost of HD and PD including travel, meals and caregiver expenditure, but excluding lost productivity (patient survey)
Beby, 2016 [31], Netherlands	Incident patients ≥ 18 years old requiring dialysis for ESRD (NR)	cICHD (SoC) iICHD cHHD iHHD	Markov, payer (2015)	5 years, 4% for costs and 1.5% for outcomes	Survival (European registry), transitioning between therapies (national registry), transplant probability (national registry), hospitalisation (published source), access failure (published sources)	EQ-5D scores from the published literature	cICHD: 0.56 iICHD: 0.61 cHHD: 0.69 iHHD: 0.75 Transplant: 0.81	Treatment initiation (published sources) Access failure (published source) Weekly tariffs (national costs) Medications (national costs) Complications (published source)	Transport (published source)
Chang, 2016 [32], Taiwan	Incident ESRD patients > 18 years receiving HD or PD (ICHD 54.3 years, PD 54.2 years)	ICHD PD	Direct measurement of monthly survival rates and average costs of medical services after initiation of dialysis, NR (2010)	12 years, 3%	Survival (national cohort)	EQ-5D scores using Taiwan value set (UK value set used in sensitivity analysis)	ICHD: 0.83 PD: 0.90	Total monthly medical costs after initiation of dialysis, including comorbidities and complications (national health insurance programme)	No
Ferguson, 2020 [39], Canada	Adult incident maintenance dialysis patients (ICHD 64.6 years, PD 61.3 years)	ICHD PD	Markov, healthcare payer (2016)	10 years, 5% for all costs and utilities	Mortality and treatment switching (national registry)	Published literature	ICHD: 0.71 PD: 0.71 HHD: 0.71 Transplant: 0.82	Direct dialysis-related and hospitalisation costs (published source)	No
Haller, 2011 [33], Austria	Individuals ≥ 18 years initiating RRT (ICHD 61.5 years, PD 47.1 years, transplant 48.1 years)	ICHD PD Transplant	Markov, public health (NR)	10 years, 3%	Survival, transition between treatments (registry)	Published literature using EQ-5D, standard gamble and TTO	ICHD 0.66 PD: 0.81 Transplant: 0.9	Monthly treatment costs, medication, non-ESRD admissions (insurance and hospital cost data)	Transportation
Howard, 2009 [34], Australia	Incident ESRD patients aged > 25 years receiving dialysis or who have a kidney transplant (NR)	ICHD HHD PD Transplant	Markov, payer (2004)	5 years, 5% for costs and outcomes	Survival, transition between treatments (national registry)	Published literature using TTO	Utility: 0.55	Direct costs of dialysis, hospitalisations, technical failure, cost of switching (published source)	No
Klarenbach, 2014 [35], Canada	Adult HD patients seeking to commence nHHD (54.1 years)	ICHD nHHD	Markov, societal (2012)	Lifetime, 5% for costs and benefits	Technique failure (RCT), mortality (national registry), transplant (AKDN)	Published EQ-5D scores	ICHD (0–6 months): 0.66 ICHD (≥ 6 months): 0.61 nHHD (0–6 months): 0.7 nHHD (≥ 6 months): 0.71	Annual dialysis costs, training and setup costs, medications (microcosting)	Patient-borne costs (microcosting)
Kontodimopoulos, 2008 [41], Greece	Prevalent RRT patients ≥ 18 years (ICHD 58.1 years, PD 58.7 years, transplant 43.7 years)	ICHD PD Transplant	Markov, payer (NR)	Lifetime, 5% for costs and benefits	Survival (ERA–EDTA)	SF-6D	Mean SF-36 scores HD: 0.639 PD: 0.599	Direct dialysis costs, including training for PD (accounts of 3 private and 2 public dialysis facilities)	No
Liu, 2015 [42], UK	Adult ESRD population requiring RRT (NR)	cICHD iICHD iHHD	Markov, payer (2013/14)	Lifetime	Survival (ERA–-EDTA), treatment switching (publication/assumptions), hospitalisations (Frequent Haemodialysis Network/assumptions)	Published literature	ICHD: 0.56 iICHD: 0.61 cHHD: 0.69 iHHD: 0.75 (published SLR/ assumptions)	Dialysis costs, hospitalisation, transplant (existing and hypothetical tariffs)	Transport (NHS costs)
Moradpour, 2020 [43], Iran	Prevalent adult patients requiring RRT (41.6 years)	HD PD Transplant	Markov, societal (2017)	Lifetime horizon, 6.0% for costs and outcomes	Treatment switching, mortality (patient hospital records)	EQ-5D-3L Persian version	HD: 0.795 PD: 0.76 Transplant: 0.945 (published sources)	Direct medical costs (hospital billing and medical records) Direct non-medical and indirect costs (patient interviews and questionnaire)	Direct non-medical costs including travel, food, and accommodation expenses and indirect costs such as caregiver income loss (patient interviews and questionnaire)
Pike, 2017 [45], Norway	Patients > 18 years with ESRD requiring dialysis (NR)	ICHD sHD scHD HHD PD	Markov, societal (2012)	5 years, 4% for costs and benefits	Mortality (SLR), transition between treatments (national registry)	Published literature	Dialysis: 0.54 Infection: -0.19 AMI: -0.27 Angina: -0.22 Sepsis: -0.28	Dialysis costs (local/national data) Training (assumption) Telemedicine (published cost) Home care (national costs)	Transport (local costs) Value of leisure time (assumption)
Surendra, 2019 [44], Malaysia	Patients > 18 years receiving dialysis (NR)	ICHD PD	Markov, payer (2017)	5 years, 3% for costs and outcomes	Survival Transition between treatments (national registry)	Published literature	ICHD: 0.854 PD: 0.905	Dialysis and ESRD management costs, including access surgery and hospitalisations (published source)	No
Treharne, 2014 [36], UK	Incident cohort of adults with ESRD requiring dialysis (NR)	ICHD PD	Markov, payer (2013/14)	5 and 10 years, 3.5% for costs and benefits	Baseline modality distribution (registry), survival (ERA–EDTA), modality switching (published data, hospital data, assumptions), all-cause hospitalisation (published sources)	Published literature	ICHD: 0.56 cHHD: 0.69 PD: 0.58 Transplant: 0.81	Access costs. dialysis services, ESA, patient monitoring, hospitalisation, transplant (national costs)	Transport
Villa, 2012 [37], Spain	Incident ESRD patients on HD or PD (NR)	HD PD	Markov, societal (2010)	5, 10 and 15 years, 3.5%	Treatment switching, mortality (registry and published sources)	SF-36 scores converted to SF-6D utilities	HD and PD: 0.69	Scheduled incidence cost, non-scheduled incidence cost, transition, prevalence cost (source not provided)	Indirect cost (source not provided)
Wong, 2020 [28], Hong Kong	Individuals requiring first-line RRT simulated at age 60 years (NA)	ICHD HHD PD	Markov, payer and societal (2017)	Lifetime, 3% for costs and outcomes	Mortality (published sources) Treatment switching (cost analysis and published sources)	Published literature	Disutilities: ICHD: 0.269 HHD: 0.222 PD: 0.222	Direct costs associated with dialysis and its delivery in first and subsequent years (published microcosting analysis)	Direct non-medical costs (e.g. patient out of pocket costs) and indirect costs (time costs) (published sources)
Wu, 2020 [46], China	Patients ≥ 18 years with ESRD receiving RRT (ICHD 49.4 years, PD 51.5 years, transplant 36.4 years)	ICHD PD Transplant	Observed cost/observed QALY = ICER (2018)	NR, NR	NR	SF-36	ICHD: 50.83 PD: 53.05	Direct medical costs (calculated)	Direct non-medical costs including transport, accommodation, nutrition; indirect costs including productivity loss (calculated)
Yang, 2016 [29], Singapore	Newly diagnosed ESRD patients aged 60 years (NA)	ICHD PD	Markov, societal, (2015)	10 years, 3% for costs and QALYs	Mortality (published source) Transplant rate (national registry)	EQ-5D	ICHD: 0.635 CAPD: 0.587 APD: 0.629	Direct medical costs (local and national data) Hospitalisation (medical records)	Transport costs (national data) Indirect costs—lost productivity for patients < 62 years only (calculated)
Yang 2021 [40], China	Patients requiring first-line RRT (HD 52.7 years, PD 41.2 years, transplant 41.8 years)	HD PD Transplant	Markov, payer (2018)	5, 10 and 15 years, 5% for costs and outcomes	Treatment switching, mortality (national registry)	KDQOL-SFV.1.3 used to generate SF-6D scores	HD: 0.61 PD: 0.61 Transplant: 0.73	Annual direct medical cost (published source)	No

^a^Summary data are provided for each study; original publication should be referred to for detail

*AKDN* Alberta Kidney Disease Network, *APD* automated peritoneal dialysis, *CAPD* continuous ambulatory peritoneal dialysis, *cHHD* conventional home haemodialysis, *cICHD* conventional in-centre haemodialysis, *ERA–EDTA* European Renal Association–European Dialysis and Transplant Association, *ESA* erythropoietin stimulating agents, *ESRD* end-stage renal disease, *HD* haemodialysis, *HHD* home haemodialysis, *ICER* incremental cost-effectiveness ratio, *ICHD* in-centre haemodialysis, *iICHD* intensive in-centre haemodialysis, *iHHD* intensive home haemodialysis, *KDQOL-SFV.1.3* kidney disease quality of life short form, *NA* not applicable, *nHHD* nocturnal home haemodialysis, *NHS* National Health Service, *NR* not reported, *PD* peritoneal dialysis, *QALY* quality-adjusted life year, *RCT* randomised controlled trial, *RRT* renal replacement therapy, *scHD* self-care haemodialysis, *sHD* satellite haemodialysis, *SF-36* short form-36, *SoC* standard of care, *SLR* systematic literature review, *TTO* time trade-off

### Model design, structure and perspective

Of the 19 included studies, 7 were conducted in Europe, 9 in Asia, 2 in Canada, and 1 in Australia (Table 2). The predominant model type was a Markov model, used in 16/19 of the included studies. The most common time horizon was a lifetime horizon (7/19 studies), with other model horizons considered including 5, 10, 12, and 15 years (Table 2). Three studies considered a range of time horizons [30, 37, 38]. Two studies conducted a cost-effectiveness analysis within a defined study period as opposed to extrapolating outcomes over a designated time horizon [33, 47]. Nine studies were conducted from a payer/health service perspective [32, 34, 35, 37, 40–43, 45], six from a societal perspective [30, 31, 36, 38, 44, 46], and two included both a payer and societal perspective [29, 39]. There was considerable variation in discount rates used across studies (1.5–6.0%), in accordance with local recommendations. Treatment switching to another modality was permitted in all but four studies [30, 33, 42, 47].

### Data inputs

The most common clinical data inputs were survival/mortality data and treatment switching, which were included in the majority of studies (Table 2). Sources of such data typically included registries and published estimates. One study included complications associated with dialysis [31] and three included data on hospitalisations [32, 37, 43]. A wide range of utility values were utilised across the studies. Utility values applied ranged from 0.54 to 0.85 for ICHD, 0.54 to 0.75 for HHD, and 0.54 to 0.905 for PD (Table 2). Five studies applied a constant utility value across each dialysis modality [35, 38, 40, 41, 46]. Only one study applied a disutility for complications that led to treatment switching [31], and one study applied disutilities for the occurrence of certain adverse events [46]. All studies included direct costs of dialysis treatment; however, the extent of what elements were covered varied somewhat between studies. Most (13/19) studies incorporated some form of non-medical direct costs or indirect costs, but these were generally not comprehensive.

### Study results

A summary of the results and reported limitations of each study is presented in Table 3. Across the 15 studies that compared ICHD/HHD with PD, PD was consistently reported to be the most cost-effective intervention. Similarly, across the 5 studies that compared HHD with ICHD, HHD was consistently reported to be the most cost-effective intervention, regardless of the frequency or duration of HHD (conventional or intensive) [32, 35, 36, 43, 46]. Only two studies assessed cost-effectiveness of HHD and PD and the results were mixed, with one study reporting that HHD was cost-effective when compared with PD [29], and the other reporting that HHD was not cost-effective when compared with PD [46].

**Table 3:** Summary of results of published dialysis cost-effectiveness studies

Study	Base case results	Sensitivity analysis results	Limitations
Afiatin, 2017 [30]	PD first: 193.2 million IDR/QALY ICHD first: 207.4 million/QALY Neither the PD- nor the ICHD-first policy provides good value for money when the ceiling ratio is similar to 43 million IDR Cost reference year: 2015	Irrespective of the willingness to pay, the HD-first policy was not cost-effective because it provided a higher cost but lower QALY than the PD-first policy	Lack of survival data for PD, which was assumed to be similar to HD, source of direct costs was hospital billing data, which has little wider (national) generalisability
Bayani, 2020 [38]	ICER for PD-first policy vs current scenario^a^ was 570,029 PHP/QALY gained ICER for PD-first option with pre-emptive transplants vs current scenario^a^ was 577,989 PHP/QALY gained ICER for expanded HD policy vs current scenario^a^ was 1,522,437 PHP/QALY gained Cost reference year: 2019	At the current threshold of 150,000 PHP, PD-first policy is the most favoured option	Some of the local data used may not be completely representative of clinical practice at a national level
Beby, 2016 [31]	Three scenarios: 1.iICHD vs ICHD: €275,747/QALY 2.cHHD vs ICHD: €12,292/QALY 3.iHHD vs ICHD: €3,248/QALY Cost reference year: 2015	Parameters with the greatest effect for each scenario and probability of cost-effectiveness were: 1.Hospital reimbursement levels and the frequency of HD; 0% probability of cost-effectiveness 2.Utilities associated with cHHD benefit and the reimbursement tariff for iHHD; 90% probability of cost-effectiveness 3.Utilities for cHHD and reimbursement tariffs for cICHD and cHHD; 58% probability of cost-effectiveness	Lack of good quality utility data, multiple treatment strategies that could be considered for iHHD
Chang, 2016 [32]	PD dominated ICHD; cost-effectiveness ratios were USD$13,681/QALY for PD and USD$16,643/QALY for ICHD Cost reference year: 2010	NA	Patients who had switched treatment were excluded, patients were matched for clinical characteristics, which may not be generalisable to the whole dialysis population, QoL values were assumed to be constant across the study, estimated costs did not include family care, and opportunity costs were not included (e.g. PD patients are more likely to be employed)
Ferguson, 2020 [39]	Cost–utility ratios vs no treatment: •ICHD: CaD$108,526.79/QALY •Home PD starters: CaD$87,126.59/QALY Cost reference year: 2016	In univariate sensitivity analyses, the cost of ICHD and the cost of an all-cause hospitalisation event were the most influential parameters Probabilistic sensitivity analysis of expected average 10-year cost + QALYs of kidney care were $349,942.93 ± $72,123.06 and 3.24 ± 0.16 QALYs for ICHD and $334,003.67 ± $53,566.75 and 3.88 ± 0.17 QALYs for PD	The model was unable to completely account for rare patient trajectories, did not account for assisted home dialysis programs or the intensity of care provided, and assumed no difference in costs between satellite dialysis units and tertiary care centre units
Haller, 2011 [33]	A 20% increase in incident patient allocation to PD saved €26 million with a discount rate of 3% and gained 839 QALYs over 10 years Cost reference year: not reported	The model was most sensitive to the cost of PD	Utility data were from published values that are several years old
Howard, 2009 [34]	Increasing the rate of PD or HHD from baseline to the highest rates observed nationally: PD saved AUD$122,061,463 and was at least as effective over 5 years HHD saved AUD$46,584,155 and was at least as effective over 5 years Cost reference year: 2004	No	Primary research was not undertaken to determine the costs and benefits of providing RRT, the analysis did not include costs of comorbidities or indirect costs
Klarenbach, 2014 [35]	nHHD dominated ICHD (0.384 incremental QALYs and -$6,668 (CaD) costs Cost reference year: 2012	nHHD was not cost-effective when the annual probability of technique failure was ≥ 19%	Small sample size and short trial duration, uncertainty around true QoL differences between modalities
Kontodimopoulos, 2008 [41]	Cost/QALY was lower for PD (€54,504) than HD (€60,353) Cost reference year: not reported	Discount rates were varied between 3 and 10% for costs and 0–5% for QALYs; in all analyses the cost/QALY was lower for PD than HD	Hospitalisation costs and indirect costs were not included in the analysis
Liu, 2015 [42]	iICHD vs ICHD: £126,106/QALY iHHD vs ICHD: dominant Cost reference year: 2013/14	For iICHD vs ICHD, costs and frequency of HD sessions had the greatest impact on the results; the probability that iICHD was cost-effective at £20,000/QALY was 0% For iHHD vs ICHD, the weekly tariff and utility of HHD had the greatest impact on the results; the probability that iHHD was cost-effective at £20,000/QALY was 97.4%	Lack of high-quality published data; utility benefit for iHHD was derived from a small study, utility decrement associated with hospitalisation was not captured, lack of UK-specific survival data
Moradpour, 2020 [43]	ICER for HD: $2,227/QALY gained ICER for PD: $1,850/QALY gained Cost reference year: 2017	The parameters that had the largest impact on the model were utility of transplantation and discounted rate for utilities At a WTP threshold of $12,380, the probability of transplantation being more cost-effective versus PD was 54.5%	Calculated QALY values from other countries were used rather than values from the country of interest, data may not be representative of all ESRD patients and there was a short follow-up period
Pike, 2017 [45]	ICER for HHD vs PD was €355,822 HHD dominated ICHD, sHD and scHD Cost reference year: 2012	PD was the most likely treatment to be cost-effective	Lack of data comparing HD modalities, lack of good quality utility data and lack of reliable cost information. Lost productivity costs were not considered
Surendra, 2019 [44]	ICER calculated based on changing uptake from baseline of 40% PD: Scenario 1 (55% HD, 45% PD): dominated Scenario 2 (50% HD, 50% PD): the most cost-effective treatment option (RM43,591) Scenario 3 (70% HD, 30% PD): dominated Cost reference year: 2017	No	Training costs for dialysis were not included
Treharne, 2014 [36]	Compared with the reference scenario (22% PD, 78% HD), increasing PD use (scenario 1: 39% PD, 61% HD and (scenario 2: 50% PD, 50% HD) was the dominant strategy at a 5 and 10 year time horizon. Both strategies dominated the third scenario (5% PD, 95% HD) at 5 and 10 years Cost reference year: 2013/14	The probability that scenario 1 was cost-effective was almost 100% at a WTP threshold of £50,000 per QALY	Data used may not be completely representative of UK clinical practice, data are only applicable to patients who are willing and able to perform PD, indirect costs were not included in the analysis
Villa, 2012 [37]	Compared with the current RRT programme (10% receiving PD), increasing the number of incident patients receiving PD to 30% was the dominant treatment strategy (ICER of –€354, 977/QALY gained) Cost reference year: 2010	ICER did not significantly change when parameters were varied ± 10%	None reported
Wong, 2020 [28]	From a payer perspective, ICHD was dominated by PD, while HHD was cost-effective versus PD (ICER USD$16,934/QALY gained) From a societal perspective ICHD was dominated by PD, while HHD was cost-effective versus PD (ICER USD$1,195/QALY gained) Cost reference year: 2017	The parameters that had the largest impact on the ICER were discount rate (payer perspective) and HHD cost after year 1 (societal perspective) At a WTP threshold of USD$18,609/QALY, the probability that PD, HHD and ICHD were cost-effective were 40, 0 and 60%, respectively, from a payer perspective, and 54, 0 and 46%, respectively, from a societal perspective	Demographic and clinical characteristics were confounded by treatment indications, potential utility differences between incident and prevalent patients were not considered, additional costs such as capital investments and overhead costs associated with dialysis were not included, findings may not be generalisable to other health systems
Wu, 2020 [46]	The cost of improving QoL by 1 unit was USD$1,373.89 for PD and USD$2,021.20 for ICHD Cost reference year: 2018	No	Study included data from only four hospitals, recorded amounts of direct non-medical costs and indirect costs were based on patients and their family members’ recollection
Yang, 2016 [29]	ICER vs CAPD was SD$96,447 for ICHD and SD$150,652 for APD for non-diabetic patients ICER vs CAPD was SD$106,281 for ICHD and SD$607,750 for APD for diabetic patients Cost reference year: 2015	The model was most sensitive to the utility value for ICHD At a threshold of $60,000/QALY, the probability of cost-effectiveness was 36.2% for CAPD, 33.2% for APD and 30.6% for ICHD	Cost of hospitalisation estimated in terms of bed charges only Costs of switching not included
Yang 2021 [47]	ICER for HD vs PD at 5 years: USD$-296,605/QALY gained (dominated) ICER for HD vs PD at 10 years: USD$-117,963/QALY gained (dominated) ICER for HD vs PD at 10 years: USD$-68,204/QALY gained (dominated) Cost reference year: 2018	The parameters that had the largest impact on the model were utility of transplant and HD and costs of HD At a WTP threshold of USD$132,900 per QALY, the probabilities of HD and PD being the optimal treatment strategy were 12.95 and 15.05%, respectively	Inclusion of transition probability parameters that may not be appropriate from a Chinese perspective, assumptions about linear trends that could undermine the predictive validity in the long term, the study did not consider indirect costs

*AUD* Australian Dollars, *CaD* Canadian Dollar, *CAPD* continuous ambulatory peritoneal dialysis, *cHHD* conventional home haemodialysis, *cICHD* conventional in-centre haemodialysis, *DSA* deterministic sensitivity analysis, *ESRD* end-stage renal disease, *HD* haemodialysis, *HHD* home haemodialysis, *ICER* incremental cost-effectiveness ratio, *ICHD* in-centre haemodialysis, *IDR* Indonesian Rupiah, *iICHD* intensive in-centre haemodialysis, *iHHD* intensive home haemodialysis, *NA* not applicable, *nHHD* nocturnal home haemodialysis, *NKTI* National Kidney and Transplant Institute, *PD* peritoneal dialysis, *PHP* Philippine Pesos, *PSA* probabilistic sensitivity analysis, *QALY* quality-adjusted life year, *QoL* quality of life, *RRT*, renal replacement therapy, *scHD* self-care haemodialysis, *sHD* satellite haemodialysis, *SoC* standard of care, *USD* United States Dollars, *WTP*, willingness to pay

^a^The current scenario assumed that 94% of patients were on HD but only 90 sessions were covered. A total of 4% will utilise PD that is fully covered by a national health insurance agency (PhilHealth) for an entire year, while only 2% undergo a transplant. Those who survived the transplant surgery received immunosuppressive therapy, but were not covered by PhilHealth

Study limitations reported by the authors included a lack of good quality clinical data [31, 43] and utility data [32, 34, 36, 43], and a lack of adequate cost information [46]. A number of studies reported a lack of consideration of some elements, which may be of importance in the overall value of the treatment, such as hospitalisations, complications, training and indirect costs, as limitations. Some studies reported concerns over applicability of their findings to a wider geographical area [29, 31, 39].

## Discussion

The majority of the studies identified in the SLR compared ICHD with PD, as these are the most common two treatment modalities used. Fewer studies included HHD as a modality option, most probably due to the relatively lower preference of this modality in recent years, and there was limited evidence comparing the cost-effectiveness of HHD versus PD. There remains to be a number of uncertainties surrounding which dialysis modalities represent the most cost-effective options for patients at different points in the care pathway.

There was considerable heterogeneity between the included studies across multiple aspects of the methodology, including time horizon, discounting, utility values, sources of clinical and economic data, and extent of clinical and economic elements included, which may have an impact on the outcomes of the analyses. Furthermore, studies were conducted from a wide range of country perspectives, where models of health care, availability of dialysis modalities and costs associated with providing health services may vary substantially. Studies were also conducted using a range of cost reference years and currencies. Taking all these factors into account, it is therefore difficult to compare studies with each other. A number of studies highlighted the lack of good quality clinical and utility data [31, 32, 34, 36, 43] and many also noted that their findings may be limited in their applicability to wider geographical settings due to the local nature of the clinical and economic inputs that were used [29, 31, 39].

Quality assessment of the included economic evaluations (provided in Supplementary Table 1) revealed that in general, the studies had well defined objectives, study design, and data collection methods. However, key modelling decisions (in particular, choice of type of economic evaluation, choice of model type, choice of discount rate and choice of variables for sensitivity analysis) were not consistently justified. Further, while results were generally clearly reported and included incremental analyses, there was variability in the extent to which individual caveats were discussed and issues relating to the generalisability of results were not often addressed.

Whilst in clinical practice, patients may switch between different treatment modalities over time according to their clinical need and personal circumstances [14–16], none of the included studies accounted for this. Most studies were cohort-level simulations representing treatments at a particular point in time, with the majority of studies considering incident patients at the start of their dialysis treatment journey, and none accounted for the fact that PD is likely to be a treatment option for a limited period of time for many patients [20, 48]. Although switching treatments was often permitted within models, no utility decrements associated with the need for a treatment switch, or additional costs associated with switching treatments were considered. Furthermore, any treatment switching effects were incorporated into the costs and outcomes for the initial treatment allocation. No studies investigated the cost-effectiveness of a switch to a different treatment modality.

Few studies noted the applicability of their results to the individual as a limitation; however, Treharne et al. (2014) highlighted that their findings concerning the cost-effectiveness of PD relative to ICHD would only be applicable to those patients who were willing and able to perform PD [37]. The same rationale also applies to HHD [13]. No studies highlighted the importance of patient choice or education in optimising clinical, economic and societal outcomes.

Previous reviews of dialysis have highlighted that there is no best dialysis modality for a patient, but rather a combination of different modalities applied at the right time for the right patient, to create an optimal treatment pathway [15, 16]. The cost-effectiveness of different modalities is therefore likely to vary over the disease course and depending on individual patient characteristics.

National guidance and public health policies have emerged that recommend or incentivise specific dialysis modalities based upon conclusions of cost-effectiveness [22–25]. A lack of clarity and education regarding the initiation of dialysis [49, 50] remains, so national policies may be adopted without proper consideration of an individual’s needs, resulting in suboptimal care. Financial incentives for specific modalities would place a hurdle to initiating other modalities at the clinician level, precluding consideration of patient choice and circumstances, and it has been reported that where there is little or no reimbursement of a modality, uptake can be low [51]. It has been noted that strong financial incentives for clinical outcomes risk undermining valued aspects of the service user–provider relationship [52]. The National Institute for Health and Care Excellence (NICE) guidelines for RRT in the UK state that the decision to start dialysis should be made jointly by the person (or, where appropriate, their family members or carers) and their healthcare team [49]. Shared decision-making between people and clinicians about their care leads to more realistic expectations, a better match between individuals’ values and treatment choices, and fewer unnecessary interventions [52, 53].

Decision-making within renal care should be holistic and comprehensive so that health outcomes are optimised for each patient and resources are allocated appropriately. Future work in this area should consider a pathway-based cost study, mapping costs to data from large care networks to more accurately reflect resource use associated with renal care.

## Conclusions

Dialysis is a life-saving intervention, but a patient’s care pathway is likely to evolve over time due to a changing lifestyle, increasing age and declining health. Switching between dialysis modalities is often essential. The cost-effectiveness of any intervention is reliant on its effectiveness, which, in the case of dialysis, is inherently linked with patient acceptability of and suitability for a particular modality. Estimates of cost-effectiveness for a particular dialysis modality at a specific point in time should be considered within the context of the holistic ESRD pathway. Each dialysis method is distinct and may be applicable to different points in care; therefore, modalities should be considered complementary and not competitively. Personalised care, including offering the right modality to the right patient at the right time, will maximise patient outcomes and minimise expenditure.

## Supplementary Information

Below is the link to the electronic supplementary material.

Supplementary file1 (DOCX 92 KB)

Supplementary file2 (XLSX 43 KB)

## Data Availability

Not applicable.

## References

[1] Stringer S (2013). The natural history of, and risk factors for, progressive chronic kidney disease (CKD): the Renal Impairment in Secondary care (RIISC) study; rationale and protocol. BMC Nephrol..

[2] The Renal Association *UK Renal Registry: 22nd Annual Report.* (2018) 04/08/2020; Available from: https://www.renalreg.org/wp-content/uploads/2020/07/22nd_UKRR_ANNUAL_REPORT_FULL.pdf

[3] Gomez AT (2015). Comorbidity burden at dialysis initiation and mortality: a cohort study.. Can. J. Kidney Health Dis..

[4] Crews DC (2014). Low income, community poverty and risk of end stage renal disease.. BMC Nephrol..

[5] The Renal Association (2011). Assessment of the potential kidney transplant recipient.

[6] NHS Choices Waiting list - kidney transplant. (2015)

[7] Deutsche Stiftung Organtransplantation. *Warteliste und Vermittlung (Waiting list and mediation)* September 2018]; Available from: https://www.dso.de/organspende-und-transplantation/warteliste-und-vermittlung.html

[8] MedTech Europe. Improving dialysis for patients and health systems in community and home care. Available from: https://www.medtecheurope.org/resource-library/improving-dialysis-for-patients-and-health-systems-in-community-and-home-care/ (2015)

[9] Levin A, Stevens PE (2014). Summary of KDIGO 2012 CKD guideline: behind the scenes, need for guidance, and a framework for moving forward. Kidney Int.

[10] Saggi SJ (2012). Considerations in the optimal preparation of patients for dialysis. Nat Rev Nephrol.

[11] Kidney Health New Zealand. *Conservative treatment* September 2018]; Available from: https://www.health.govt.nz/system/files/documents/topic_sheets/conservative-treatment.pdf

[12] Blake PG, Quinn RR, Oliver MJ (2013). Peritoneal dialysis and the process of modality selection. Perit Dial Int.

[13] Rioux JP (2015). Patient selection and training for home hemodialysis. Hemodial Int.

[14] Covic A (2010). Educating end-stage renal disease patients on dialysis modality selection: clinical advice from the European Renal Best Practice (ERBP) Advisory Board. Nephrol Dial Transplant.

[15] Imbeault B, Nadeau-Fredette AC (2019). Optimization of dialysis modality transitions for improved patient care. Can J Kidney Health Dis.

[16] Lambie M, Davies SJ (2015). Transition between home dialysis modalities: another piece in the jigsaw of the integrated care pathway. Nephrol Dial Transplant.

[17] Diaz-Buxo JA, White SA, Himmele R (2013). The importance of residual renal function in peritoneal dialysis patients. Adv Perit Dial.

[18] Salzer WL (2018). Peritoneal dialysis-related peritonitis: challenges and solutions. Int J Nephrol Renovasc Dis.

[19] Struijk DG (2008). Monitoring of the peritoneal membrane NDT Plus. Clin Kidney.

[20] Mayo Clinic. *Peritoneal dialysis*. 2021 July 2021]; Available from: https://www.mayoclinic.org/tests-procedures/peritoneal-dialysis/about/pac-20384725

[21] European Kidney Health Alliance. *Recommendations for Sustainable Kidney Care*. 2015 July 2021]; Available from: http://ekha.eu/wp-content/uploads/2016/01/EKHA-Recs-for-Sustainable-Kidney-Care-25.08.2015.pdf

[22] Hager D, Ferguson TW, Komenda P (2019). Cost controversies of a "Home Dialysis First" Policy. Can J Kidney Health Dis..

[23] Teerawattananon Y (2016). How to meet the demand for good quality renal dialysis as part of universal health coverage in resource-limited settings?. Health Res Policy Syst.

[24] Teerawattananon Y, Mugford M, Tangcharoensathien V (2007). Economic evaluation of palliative management versus peritoneal dialysis and haemodialysis for end-stage renal disease: evidence for coverage decisions in Thailand. Value in Health.

[25] Li PK, Chow KM (2001). The cost barrier to peritoneal dialysis in the developing world–an Asian perspective. Perit Dial Int.

[26] NHS Kidney Care, *Transplant first: timely listing for kidney transplantation.* Available from: https://www.england.nhs.uk/improvement-hub/wp-content/uploads/sites/44/2017/11/Transplant-First-Timely-Listing-for-Kidney-Transplantation.pdf (2013)

[27] Page MJ (2021). The PRISMA 2020 statement: an updated guideline for reporting systematic reviews. BMJ.

[28] Drummond MF, Jefferson TO (1996). Guidelines for authors and peer reviewers of economic submissions to the BMJ. BMJ.

[29] Wong CKH (2020). Lifetime cost-effectiveness analysis of first-line dialysis modalities for patients with end-stage renal disease under peritoneal dialysis first policy. BMC Nephrol.

[30] Yang F, Lau T, Luo N (2016). Cost-effectiveness of haemodialysis and peritoneal dialysis for patients with end-stage renal disease in Singapore. Nephrology (Carlton).

[31] Afiatin,  (2017). Economic evaluation of policy options for dialysis in end-stage renal disease patients under the universal health coverage in Indonesia. PLoS One.

[32] Beby AT (2016). Cost-effectiveness of high dose hemodialysis in comparison to conventional in-center hemodialysis in the Netherlands. Adv Ther.

[33] Chang YT (2016). Cost-effectiveness of hemodialysis and peritoneal dialysis: a national cohort study with 14 years follow-up and matched for comorbidities and propensity score. Sci Rep.

[34] Haller M (2011). Cost-effectiveness analysis of renal replacement therapy in Austria. Nephrol Dial Transplant.

[35] Howard K (2009). The cost-effectiveness of increasing kidney transplantation and home-based dialysis. Nephrology (Carlton).

[36] Klarenbach S (2014). Economic evaluation of frequent home nocturnal hemodialysis based on a randomized controlled trial. J Am Soc Nephrol.

[37] Treharne C (2014). Peritoneal dialysis and in-centre haemodialysis: a cost–utility analysis from a UK payer perspective. Appl Health Econ Health Policy.

[38] Villa G (2012). Cost-effectiveness analysis of the Spanish renal replacement therapy program. Perit Dial Int.

[39] Bayani DBS (2021). Filtering for the best policy: An economic evaluation of policy options for kidney replacement coverage in the Philippines. Nephrology (Carlton).

[40] Ferguson TW (2021). Cost-utility of dialysis in Canada: hemodialysis, peritoneal dialysis, and nondialysis treatment of kidney failure. Kidney Med.

[41] Yang F (2021). Cost-effectiveness analysis of renal replacement therapy strategies in Guangzhou city, southern China. BMJ Open.

[42] Kontodimopoulos N, Niakas D (2008). An estimate of lifelong costs and QALYs in renal replacement therapy based on patients' life expectancy. Health Policy.

[43] Liu FX (2015). High-dose hemodialysis versus conventional in-center hemodialysis: a cost–utility analysis from a UK payer perspective. Value Health.

[44] Moradpour A, Hadian M, Tavakkoli M (2020). Economic evaluation of end stage renal disease treatments in Iran. Clin Epidemiol Glob Health.

[45] Surendra NK (2019). Cost utility analysis of end stage renal disease treatment in ministry of health dialysis centres, Malaysia: hemodialysis versus continuous ambulatory peritoneal dialysis. PLoS ONE.

[46] Pike E (2017). More use of peritoneal dialysis gives significant savings: a systematic review and health economic decision model. J Clin Med Res.

[47] Wu H (2020). Economic burden and cost–utility analysis of three renal replacement therapies in ESRD patients from Yunnan Province, China.. Int Urol Nephrol.

[48] Rydell H (2019). Fewer hospitalizations and prolonged technique survival with home hemodialysis- a matched cohort study from the Swedish renal registry. BMC Nephrol.

[49] National Institute for Health and Care Excellence. *NG107: Renal replacement therapy and conservative management.* 2018 27/08/2020]; Available from: https://www.nice.org.uk/guidance/NG107 31194310

[50] CADTH (Canadian Agency for Drugs and Technologies in Health) Dialysis modalities for the treatment of end-stage kidney disease: a health technology assessment. Optimal Use Report **6**(2b), 14–15 (2017) 30325617

[51] Just PM (2008). Reimbursement and economic factors influencing dialysis modality choice around the world. Nephrol Dial Transplant.

[52] Systems A-C (2020). Evidence, Strategies and Challenges.

[53] NHS England. *What is personalised care?* 2020 04/08/2020]; Available from: https://www.england.nhs.uk/personalisedcare/what-is-personalised-care/

