# Supplementary material for: A systematic review of the cost-effectiveness of renal replacement therapies, and consequences for decision-making in the end-stage renal disease treatment pathway
Source: Eur J Health Econ. 2022 Jun 18;24(3):377–92. doi: 10.1007/s10198-022-01478-2 (PMC10060297; doi:10.1007/s10198-022-01478-2)
Supplement: Supplementary file 1 — Supplementary file1 (DOCX 92 KB) [file 10198_2022_1478_MOESM1_ESM.docx]

**Article title:** A systematic review of the cost-effectiveness of renal replacement therapies, and consequences for decision making in the end-stage renal disease treatment pathway

**Journal name:** European Journal of Health Economics

**Authors:** Ellen Busink MLitt MSc1, Dana Kendzia MSc1, Fatih Kircelli MD2, Sophie Boeger MSc1, Jovana Petrovic MSc1, Helen Smethurst PhD3, Stephen Mitchell PhD3, Christian Apel MSc1

**Affiliations:** 1.Health Economics, Market Access & Political Affairs, Fresenius Medical Care, Bad Homburg, Germany; 2. Global Medical Information & Education, Fresenius Medical Care, Bad Homburg, Germany; 3. Mtech Access Ltd, Bicester, UK

**Corresponding author:** Ellen Busink, Fresenius Medical Care, Else-Kröner-Straße 3, 61352 Bad Homburg, +49 6172 609 2304, [ellen.busink@fmc-ag.com](mailto:ellen.busink@fmc-ag.com)

**Supplementary information**

S1. Search strategies for original SLR

Embase 1974 to 2020 March 02: accessed March 3rd 2020

| **#** | **Searches** | **Results** |
| --- | --- | --- |
| 1 | Kidney Failure, Chronic/ | 18057 |
| 2 | (CKF or CKD or CRF or CRD).mp. | 73430 |
| 3 | (predialysis or pre-dialysis).mp. | 7156 |
| 4 | (chronic kidney or chronic renal).mp. | 168048 |
| 5 | ((endstage or end-stage) adj2 kidney).mp. | 5762 |
| 6 | ((endstage or end-stage) adj2 renal).mp. | 69202 |
| 7 | (ESRF or ESKF or ESRD or ESKD).mp. | 29473 |
| 8 | 1 or 2 or 3 or 4 or 5 or 6 or 7 | 250936 |
| 9 | (cost minimi?ation analys* or (cost-minimi?ation adj1 analys*)).mp. | 3815 |
| 10 | exp Cost-Benefit Analysis/ | 83511 |
| 11 | ((cost benefit adj1 analys*) or (cost-benefit adj1 analys*)).mp. | 85892 |
| 12 | (cost utility analys* or (cost-utility adj1 analys*)).mp. | 10650 |
| 13 | (cost consequence analys* or (cost-conseq* adj1 analys*)).mp. | 411 |
| 14 | ((cost-effective* adj1 analys*) or "cost adj1 effectiveness adj1 analys*").mp. | 151866 |
| 15 | 9 or 10 or 11 or 12 or 13 or 14 | 231340 |
| 16 | ((economic or pharmacoeconomic) adj1 (evaluation or assessment or analys?s or stud*)).mp. | 34961 |
| 17 | ("CEA" or "CMA" or "CBA" or "CUA" or "CCA").mp. | 70057 |
| 18 | exp decision theory/ or exp decision trees/ | 14049 |
| 19 | decision tree.mp. | 16199 |
| 20 | models, economic/ | 1955 |
| 21 | (markov or deterministic).mp. | 46272 |
| 22 | ((transition adj1 probabilit*) or (health adj1 stat*) or (sensitivity adj1 analys*) or (health adj1 outcome)).mp. | 319277 |
| 23 | ((patient level or patient-level or discrete event or discrete-event) adj1 simulat*).mp. | 1332 |
| 24 | (incremental-cost or incremental cost).mp. | 18472 |
| 25 | (ICER or QALY or DALY or WTP or TTO).mp. | 25274 |
| 26 | 17 or 18 or 19 or 20 or 21 or 22 or 23 or 24 or 25 | 453253 |
| 27 | 16 and 26 | 11478 |
| 28 | 15 or 27 | 233872 |
| 29 | exp continuous ambulatory peritoneal dialysis/ or dialysis/ or exp peritoneal dialysis/ or exp continuous ambulatory peritoneal dialysis system/ or exp extended daily dialysis/ or exp automated peritoneal dialysis system/ or exp home dialysis/ | 86762 |
| 30 | (h?emodialysis or dialysis).mp. | 248329 |
| 31 | (HHD or HD or PD or ICHD).mp. | 2227552 |
| 32 | 29 or 30 or 31 | 2435379 |
| 33 | 8 and 28 and 32 | 1368 |

Ovid MEDLINE(R) and Epub Ahead of Print, In-Process & Other Non-Indexed Citations, Daily and Versions(R) 1946 to March 02, 2020: accessed March 3rd 2020

| **#** | **Searches** | **Results** |
| --- | --- | --- |
| 1 | Kidney Failure, Chronic/ | 92301 |
| 2 | (CKF or CKD or CRF or CRD).mp. | 46502 |
| 3 | (predialysis or pre-dialysis).mp. | 4976 |
| 4 | (chronic kidney or chronic renal).mp. | 83897 |
| 5 | ((endstage or end-stage) adj2 kidney).mp. | 3916 |
| 6 | ((endstage or end-stage) adj2 renal).mp. | 38995 |
| 7 | (ESRF or ESKF or ESRD or ESKD).mp. | 18222 |
| 8 | 1 or 2 or 3 or 4 or 5 or 6 or 7 | 182182 |
| 9 | (cost minimi?ation analys* or (cost-minimi?ation adj1 analys*)).mp. | 722 |
| 10 | exp Cost-Benefit Analysis/ | 79608 |
| 11 | ((cost benefit adj1 analys*) or (cost-benefit adj1 analys*)).mp. | 81839 |
| 12 | (cost utility analys* or (cost-utility adj1 analys*)).mp. | 3018 |
| 13 | (cost consequence analys* or (cost-conseq* adj1 analys*)).mp. | 255 |
| 14 | ((cost-effective* adj1 analys*) or "cost adj1 effectiveness adj1 analys*").mp. | 12420 |
| 15 | 10 or 11 or 12 or 13 or 14 | 86363 |
| 16 | ((economic or pharmacoeconomic) adj1 (evaluation or assessment or analys?s or stud*)).mp. | 18369 |
| 17 | ("CEA" or "CMA" or "CBA" or "CUA" or "CCA").mp. | 62031 |
| 18 | exp decision theory/ or exp decision trees/ | 11832 |
| 19 | decision tree.mp. | 7007 |
| 20 | models, economic/ | 9846 |
| 21 | (markov or deterministic).mp. | 39428 |
| 22 | ((transition adj1 probabilit*) or (health adj1 stat*) or (sensitivity adj1 analys*) or (health adj1 outcome)).mp. | 207850 |
| 23 | ((patient level or patient-level or discrete event or discrete-event) adj1 simulat*).mp. | 785 |
| 24 | (incremental-cost or incremental cost).mp. | 11096 |
| 25 | (ICER or QALY or DALY or WTP or TTO).mp. | 14103 |
| 26 | 18 or 19 or 20 or 21 or 22 or 23 or 24 or 25 or "37".mp. | 678355 |
| 27 | 16 and 26 | 6745 |
| 28 | 15 or 27 | 88412 |
| 29 | exp Renal Dialysis/ or exp Dialysis/ or exp Peritoneal Dialysis, Continuous Ambulatory/ or exp Peritoneal Dialysis/ | 134568 |
| 30 | (h?emodialysis or dialysis).mp. | 180918 |
| 31 | (HHD or HD or PD or ICHD).mp. | 167741 |
| 32 | 29 or 30 or 31 | 340356 |
| 33 | 8 and 28 and 32 | 514 |

EBM Reviews - Cochrane Database of Systematic Reviews 2005 to February 27, 2020,  EBM Reviews - ACP Journal Club 1991 to February 2020,  EBM Reviews - Database of Abstracts of Reviews of Effects 1st Quarter 2016,  EBM Reviews - Cochrane Clinical Answers February 2020,  EBM Reviews - Cochrane Central Register of Controlled Trials January 2020,  EBM Reviews - Cochrane Methodology Register 3rd Quarter 2012,  EBM Reviews - Health Technology Assessment 4th Quarter 2016, EBM Reviews - NHS Economic Evaluation Database 1st Quarter 2016: accessed March 3^rd^ 2020

| **#** | **Searches** | **Results** |
| --- | --- | --- |
| 1 | Kidney Failure, Chronic/ | 4786 |
| 2 | (CKF or CKD or CRF or CRD).mp. | 53960 |
| 3 | (predialysis or pre-dialysis).mp. | 1030 |
| 4 | (chronic kidney or chronic renal).mp. | 11203 |
| 5 | ((endstage or end-stage) adj2 kidney).mp. | 764 |
| 6 | ((endstage or end-stage) adj2 renal).mp. | 4875 |
| 7 | (ESRF or ESKF or ESRD or ESKD).mp. | 2444 |
| 8 | 1 or 2 or 3 or 4 or 5 or 6 or 7 | 66835 |
| 9 | (cost minimi?ation analys* or (cost-minimi?ation adj1 analys*)).mp. | 846 |
| 10 | exp Cost-Benefit Analysis/ | 18958 |
| 11 | ((cost benefit adj1 analys*) or (cost-benefit adj1 analys*)).mp. | 22457 |
| 12 | (cost utility analys* or (cost-utility adj1 analys*)).mp. | 4822 |
| 13 | (cost consequence analys* or (cost-conseq* adj1 analys*)).mp. | 1794 |
| 14 | ((cost-effective* adj1 analys*) or "cost adj1 effectiveness adj1 analys*").mp. | 20984 |
| 15 | 10 or 11 or 12 or 13 or 14 | 35777 |
| 16 | ((economic or pharmacoeconomic) adj1 (evaluation or assessment or analys?s or stud*)).mp. | 25542 |
| 17 | ("CEA" or "CMA" or "CBA" or "CUA" or "CCA").mp. | 5653 |
| 18 | exp decision theory/ or exp decision trees/ | 925 |
| 19 | decision tree.mp. | 1824 |
| 20 | models, economic/ | 1551 |
| 21 | (markov or deterministic).mp. | 5451 |
| 22 | ((transition adj1 probabilit*) or (health adj1 stat*) or (sensitivity adj1 analys*) or (health adj1 outcome)).mp. | 45794 |
| 23 | ((patient level or patient-level or discrete event or discrete-event) adj1 simulat*).mp. | 157 |
| 24 | (incremental-cost or incremental cost).mp. | 8404 |
| 25 | (ICER or QALY or DALY or WTP or TTO).mp. | 7394 |
| 26 | 18 or 19 or 20 or 21 or 22 or 23 or 24 or 25 or "37".mp. | 99454 |
| 27 | 16 and 26 | 13882 |
| 28 | 15 or 27 | 37117 |
| 29 | exp Renal Dialysis/ or exp Dialysis/ or exp Peritoneal Dialysis, Continuous Ambulatory/ or exp Peritoneal Dialysis/ | 5497 |
| 30 | (h?emodialysis or dialysis).mp. | 19380 |
| 31 | (HHD or HD or PD or ICHD).mp. | 41899 |
| 32 | 29 or 30 or 31 | 58090 |
| 33 | 8 and 28 and 32 | 498 |

Econlit 1886 to February 20, 2020: accessed March 3^rd^ 2020

| **#** | **Searches** | **Results** | **Type** |
| --- | --- | --- | --- |
| 1 | (h?emodialysis or dialysis).mp. | 105 | Advanced |

**S2. Search strategies for SLR update**

Embase 1974 to 2021 July 02

| **#** | **Searches** | **Results** |
| --- | --- | --- |
| 1 | Kidney Failure, Chronic/ | 56548 |
| 2 | (CKF or CKD or CRF or CRD).mp. | 89216 |
| 3 | (predialysis or pre-dialysis).mp. | 8092 |
| 4 | (chronic kidney or chronic renal).mp. | 193475 |
| 5 | ((endstage or end-stage) adj2 kidney).mp. | 8038 |
| 6 | ((endstage or end-stage) adj2 renal).mp. | 80849 |
| 7 | (ESRF or ESKF or ESRD or ESKD).mp. | 35486 |
| 8 | 1 or 2 or 3 or 4 or 5 or 6 or 7 | 290444 |
| 9 | (cost minimi?ation analys* or (cost-minimi?ation adj1 analys*)).mp. | 4036 |
| 10 | exp Cost-Benefit Analysis/ | 87390 |
| 11 | ((cost benefit adj1 analys*) or (cost-benefit adj1 analys*)).mp. | 89989 |
| 12 | (cost utility analys* or (cost-utility adj1 analys*)).mp. | 11755 |
| 13 | (cost consequence analys* or (cost-conseq* adj1 analys*)).mp. | 476 |
| 14 | ((cost-effective* adj1 analys*) or "cost adj1 effectiveness adj1 analys*").mp. | 164068 |
| 15 | 9 or 10 or 11 or 12 or 13 or 14 | 247438 |
| 16 | ((economic or pharmacoeconomic) adj1 (evaluation or assessment or analys?s or stud*)).mp. | 38700 |
| 17 | ("CEA" or "CMA" or "CBA" or "CUA" or "CCA").mp. | 75568 |
| 18 | exp decision theory/ or exp decision trees/ | 16934 |
| 19 | decision tree.mp. | 19497 |
| 20 | models, economic/ | 2463 |
| 21 | (markov or deterministic).mp. | 52597 |
| 22 | ((transition adj1 probabilit*) or (health adj1 stat*) or (sensitivity adj1 analys*) or (health adj1 outcome)).mp. | 354061 |
| 23 | ((patient level or patient-level or discrete event or discrete-event) adj1 simulat*).mp. | 1556 |
| 24 | (incremental-cost or incremental cost).mp. | 20956 |
| 25 | (ICER or QALY or DALY or WTP or TTO).mp. | 28757 |
| 26 | 17 or 18 or 19 or 20 or 21 or 22 or 23 or 24 or 25 | 503074 |
| 27 | 16 and 26 | 12937 |
| 28 | 15 or 27 | 250332 |
| 29 | exp continuous ambulatory peritoneal dialysis/ or dialysis/ or exp peritoneal dialysis/ or exp continuous ambulatory peritoneal dialysis system/ or exp extended daily dialysis/ or exp automated peritoneal dialysis system/ or exp home dialysis/ | 95589 |
| 30 | (h?emodialysis or dialysis).mp. | 274234 |
| 31 | (HHD or HD or PD or ICHD).mp. | 2359058 |
| 32 | 29 or 30 or 31 | 2587079 |
| 33 | 8 and 28 and 32 | 1503 |
| 34 | limit 33 to yr="2020 -Current" | 136 |

Ovid MEDLINE(R) and Epub Ahead of Print, In-Process, In-Data-Review & Other Non-Indexed Citations, Daily and Versions(R) 1946 to July 02, 2021

| **#** | **Searches** | **Results** |
| --- | --- | --- |
| 1 | Kidney Failure, Chronic/ | 95975 |
| 2 | (CKF or CKD or CRF or CRD).mp. | 53480 |
| 3 | (predialysis or pre-dialysis).mp. | 5305 |
| 4 | (chronic kidney or chronic renal).mp. | 94699 |
| 5 | ((endstage or end-stage) adj2 kidney).mp. | 5186 |
| 6 | ((endstage or end-stage) adj2 renal).mp. | 42143 |
| 7 | (ESRF or ESKF or ESRD or ESKD).mp. | 20233 |
| 8 | 1 or 2 or 3 or 4 or 5 or 6 or 7 | 199376 |
| 9 | (cost minimi?ation analys* or (cost-minimi?ation adj1 analys*)).mp. | 791 |
| 10 | exp Cost-Benefit Analysis/ | 85303 |
| 11 | ((cost benefit adj1 analys*) or (cost-benefit adj1 analys*)).mp. | 87812 |
| 12 | (cost utility analys* or (cost-utility adj1 analys*)).mp. | 3450 |
| 13 | (cost consequence analys* or (cost-conseq* adj1 analys*)).mp. | 291 |
| 14 | ((cost-effective* adj1 analys*) or "cost adj1 effectiveness adj1 analys*").mp. | 13970 |
| 15 | 10 or 11 or 12 or 13 or 14 | 92776 |
| 16 | ((economic or pharmacoeconomic) adj1 (evaluation or assessment or analys?s or stud*)).mp. | 20787 |
| 17 | ("CEA" or "CMA" or "CBA" or "CUA" or "CCA").mp. | 65866 |
| 18 | exp decision theory/ or exp decision trees/ | 12499 |
| 19 | decision tree.mp. | 8734 |
| 20 | models, economic/ | 10636 |
| 21 | (markov or deterministic).mp. | 44279 |
| 22 | ((transition adj1 probabilit*) or (health adj1 stat*) or (sensitivity adj1 analys*) or (health adj1 outcome)).mp. | 230634 |
| 23 | ((patient level or patient-level or discrete event or discrete-event) adj1 simulat*).mp. | 920 |
| 24 | (incremental-cost or incremental cost).mp. | 12766 |
| 25 | (ICER or QALY or DALY or WTP or TTO).mp. | 16370 |
| 26 | 18 or 19 or 20 or 21 or 22 or 23 or 24 or 25 or "37".mp. | 742483 |
| 27 | 16 and 26 | 7695 |
| 28 | 15 or 27 | 95067 |
| 29 | exp Renal Dialysis/ or exp Dialysis/ or exp Peritoneal Dialysis, Continuous Ambulatory/ or exp Peritoneal Dialysis/ | 139883 |
| 30 | (h?emodialysis or dialysis).mp. | 191329 |
| 31 | (HHD or HD or PD or ICHD).mp. | 193685 |
| 32 | 29 or 30 or 31 | 375244 |
| 33 | 8 and 28 and 32 | 560 |
| 34 | limit 33 to yr="2020 -Current" | 40 |

EBM Reviews - Cochrane Database of Systematic Reviews 2005 to June 30, 2021, EBM Reviews - ACP Journal Club 1991 to June 2021,  EBM Reviews - Database of Abstracts of Reviews of Effects 1st Quarter 2016,  EBM Reviews - Cochrane Clinical Answers June 2021,  EBM Reviews - Cochrane Central Register of Controlled Trials May 2021, EBM Reviews - Cochrane Methodology Register 3rd Quarter 2012, EBM Reviews - Health Technology Assessment 4th Quarter 2016, EBM Reviews - NHS Economic Evaluation Database 1st Quarter 2016

| **#** | **Searches** | **Results** |
| --- | --- | --- |
| 1 | Kidney Failure, Chronic/ | 4993 |
| 2 | (CKF or CKD or CRF or CRD).mp. | 55662 |
| 3 | (predialysis or pre-dialysis).mp. | 1155 |
| 4 | (chronic kidney or chronic renal).mp. | 12966 |
| 5 | ((endstage or end-stage) adj2 kidney).mp. | 970 |
| 6 | ((endstage or end-stage) adj2 renal).mp. | 5574 |
| 7 | (ESRF or ESKF or ESRD or ESKD).mp. | 2814 |
| 8 | 1 or 2 or 3 or 4 or 5 or 6 or 7 | 70012 |
| 9 | (cost minimi?ation analys* or (cost-minimi?ation adj1 analys*)).mp. | 871 |
| 10 | exp Cost-Benefit Analysis/ | 19513 |
| 11 | ((cost benefit adj1 analys*) or (cost-benefit adj1 analys*)).mp. | 23385 |
| 12 | (cost utility analys* or (cost-utility adj1 analys*)).mp. | 5076 |
| 13 | (cost consequence analys* or (cost-conseq* adj1 analys*)).mp. | 1823 |
| 14 | ((cost-effective* adj1 analys*) or "cost adj1 effectiveness adj1 analys*").mp. | 22644 |
| 15 | 10 or 11 or 12 or 13 or 14 | 38061 |
| 16 | ((economic or pharmacoeconomic) adj1 (evaluation or assessment or analys?s or stud*)).mp. | 26346 |
| 17 | ("CEA" or "CMA" or "CBA" or "CUA" or "CCA").mp. | 6668 |
| 18 | exp decision theory/ or exp decision trees/ | 929 |
| 19 | decision tree.mp. | 1965 |
| 20 | models, economic/ | 1571 |
| 21 | (markov or deterministic).mp. | 5771 |
| 22 | ((transition adj1 probabilit*) or (health adj1 stat*) or (sensitivity adj1 analys*) or (health adj1 outcome)).mp. | 49689 |
| 23 | ((patient level or patient-level or discrete event or discrete-event) adj1 simulat*).mp. | 174 |
| 24 | (incremental-cost or incremental cost).mp. | 9072 |
| 25 | (ICER or QALY or DALY or WTP or TTO).mp. | 8065 |
| 26 | 18 or 19 or 20 or 21 or 22 or 23 or 24 or 25 or "37".mp. [mp=ti, ab, tx, kw, ct, ot, sh, hw] | 109090 |
| 27 | 16 and 26 | 14208 |
| 28 | 15 or 27 | 39484 |
| 29 | exp Renal Dialysis/ or exp Dialysis/ or exp Peritoneal Dialysis, Continuous Ambulatory/ or exp Peritoneal Dialysis/ | 5736 |
| 30 | (h?emodialysis or dialysis).mp. | 21622 |
| 31 | (HHD or HD or PD or ICHD).mp. | 46530 |
| 32 | 29 or 30 or 31 | 64438 |
| 33 | 8 and 28 and 32 | 516 |
| 34 | limit 33 to yr="2020 -Current" [Limit not valid in DARE; records were retained] | 31 |

Econlit 1886 to June 24, 2021

| **#** | **Searches** | **Results** |
| --- | --- | --- |
| 1 | (h?emodialysis or dialysis).mp. | 123 |
| 2 | limit 1 to yr="2020 -Current" | 17 |

Supplemental Table. Quality assessment of included studies (Drummond checklist)
